# Caffeic Acid in Tobacco Root Exudate Defends Tobacco Plants From Infection by *Ralstonia solanacearum*

**DOI:** 10.3389/fpls.2021.690586

**Published:** 2021-08-12

**Authors:** Shili Li, Jing Pi, Hongjiang Zhu, Liang Yang, Xingguo Zhang, Wei Ding

**Affiliations:** ^1^Laboratory of Natural Products Pesticides, College of Plant Protection, Southwest University, Chongqing, China; ^2^Key Laboratory of Horticulture Science for Southern Mountainous Regions, Southwest University, Chongqing, China

**Keywords:** caffeic acid, phenolic acids, antibacterial activity, tobacco bacterial wilt, *Ralstonia solanacearum*

## Abstract

In rhizospheres, chemical barrier-forming natural compounds play a key role in preventing pathogenic bacteria from infecting plant roots. Here, we sought to identify specific phenolic exudates in tobacco (*Nicotiana tobaccum*) plants infected by the soil-borne pathogen *Ralstonia solanacearum* that may exhibit antibacterial activity and promote plant resistance against pathogens. Among detected phenolic acids, only caffeic acid was significantly induced in infected plants by *R. solanacearum* relative to healthy plants, and the concentration of caffeic acid reached 1.95 μg/mL. *In vivo*, caffeic acid at 200 μg/mL was highly active against *R. solanacearum* and obviously damaged the membrane structure of the *R. solanacearum* cells, resulting in the thinning of the cell membrane and irregular cavities in cells. Moreover, caffeic acid significantly inhibited biofilm formation by repressing the expression of the *lecM* and *epsE* genes. *In vitro*, caffeic acid could effectively activate phenylalanine ammonia-lyase (PAL) and peroxidase (POD) and promote the accumulation of lignin and hydroxyproline. In pot and field experiments, exogenous applications of caffeic acid significantly reduced and delayed the incidence of tobacco bacterial wilt. Taken together, all these results suggest that caffeic acid played a crucial role in defending against *R. solanacearum* infection and was a potential and effective antibacterial agent for controlling bacterial wilt.

## Introduction

Plants suffer from various abiotic and biotic stresses during the growth process. To adapt to adverse situations, plants have evolved the ability to perceive different stress signals from their surroundings, integrate these signals, and respond to different stresses (Verma et al., 2016). Among these adaptations, root secretion is one of the fundamental adaptive responses to environmental stresses. Specifically, plant roots produce a variety of bioactive compounds, many of which are capable of repelling, inhibiting, or killing pathogenic microorganisms (Haichar et al., 2014). Studies have clearly demonstrated that secondary metabolites secreted by plant roots are one of the strategies of plant defense and represent a protective chemical barrier (Bais et al., 2002). In general, root-secreted compounds including indoles, phenolics, terpenoids, flavonoids, benzoxazinones, and coumarins, have strong external antibacterial qualities (Baetz and Martinoia, 2014). For instance, coumarins could have obvious bacteriostatic properties and suppress the colonization and proliferation of *Ralstonia solanacearum* in tobacco roots and stems (Yang et al., 2017). Moreover, derivatives of isoflavonoids, such as phytoalexin pisatin, show potent antimicrobial properties in legumes (Wu et al., 2008). Bais et al. (2005) reported that the exudation of root-derived antimicrobial compounds by *Arabidopsis thaliana* confers tissue-specific resistance to a wide range of bacterial pathogens, and the ability of *Pseudomonas syringae* to cause disease depends on its colonization in roots and overcoming antimicrobial exudations.

Phenolic acid is a secondary metabolite derived from phenylpropionic acid, which occurs in defensive root exudates, and has ecological significance in resisting adverse environmental stress and resisting external interference factors (Mandal et al., 2010). Phenolic metabolites of plants have shown strong antimicrobial activity and exhibit important prospects in plant defense (Lanoue et al., 2010b). For instance, rosmarinic acid showed antimicrobial activity against *Aspergillus niger* through disrupting interseptas and curling cell surfaces (Bais et al., 2002). Similarly, some experiments demonstrated that coumaric and syringic acids have significant virulence on the genus *Pectobacterium* by reducing pectolytic and proteolytic exoenzyme activities (Joshi et al., 2010). Other substances such as gallic acid and caffeic acid have been reported to have antibiotic activity against *Candida tropicalis* and *Xylella fastidiosa* (Maddox et al., 2010; Lima et al., 2016). In addition, when the plant is under attack, the levels of plant phenolic acids could be largely induced to disarm the pathogen. For instance, a barley root system under *Fusarium* attack secretes phenolic compounds with antimicrobial activity, including *t*-cinnamic, *p*-coumaric, ferulic, syringic, and vanillic acids (Lanoue et al., 2010a). Among these phenolics, *t*-cinnamic acid is biosynthesized *de novo* and accumulates significantly, playing an active and dynamic role in plant defense (Lanoue et al., 2010a). Moreover, phenolic acids affect the activity of soil microbes by inhibiting or promoting the growth of some microbes *in vitro* (Li et al., 2017b). Taken together, research demonstrates that phenolic acids are an important factor in the development of plant resistance to protect plants from invaders.

*Ralstonia solanacearum* is a Gram-negative soil-borne pathogen that causes bacterial wilt disease and leads to destructive losses of some economic crops, such as potato, eggplant, tomato, peanut, and tobacco (Salanoubat et al., 2002). The use of chemical pesticides and breeding resistant varieties are the primary contemporary methods for controlling bacterial wilt. However, the long-term use of chemical pesticides results in serious environmental problems and the accumulation of residues in crops. Therefore, the search for new agents that are efficient and environmentally friendly necessarily continues (Becker and Schwinn, 1993). Based on research, the application of natural products extracted from or secreted from plants to control bacterial wilt is an effective and promising biological control method (Hassan et al., 2009). The products possess ideal inhibitory activity and include benzoxazinone derivates, coumarins, and phenols, many of which can alleviate the severity of bacterial wilt (Lowe et al., 2015; Guo et al., 2016; Yang et al., 2017). Consequently, for the control of bacterial wilt, the development of new alternatives or effective antimicrobial agents is urgent. Therefore, we investigated the potential ability of tobacco roots to secrete antimicrobial phenolic acids into the rhizosphere when challenged by *R. solanacearum*. Furthermore, we assessed the antibacterial activity of caffeic acid against *R. solanacearum* and the effect on plant defense for controlling tobacco bacterial wilt. Thus, this report provides useful information for understanding the function of phenolic acids secreted by plant roots that establish a chemical barrier around the roots that represses the growth of the pathogen and contributes to plant defense against microbial disease.

## Materials and Methods

### Bacterial Strains, Culture Conditions, and Chemicals

The *R. solanacearum* strain CQPS-1 (phylotype I, race 1, and biovar 3) was used in this study (Liu et al., 2017). *R. solanacearum* was routinely grown on nutrient agar (NA) medium and nutrient broth (NB) fluid medium (Li et al., 2017b). The standard phenolic acids used in this study included caffeic acid, *p*-coumaric acid, cinnamic acid, ferulic acid, vanillic acid, and benzoic acid. All organic acids were purchased from Sigma-Aldrich (Shanghai, China).

### Collection of Tobacco Root Exudates

The tobacco cultivar Yunyan 87 was used in this study. Seeds were surface-sterilized with 75% ethanol by gently shaking for 30 s, soaked in 3% NaClO for 10 min, and then washed five times with sterile water. The seeds were placed on quartz sand with 1/4 Murashige and Skoog (MS) solution and watered every 2 days with a 16 h light/8 h dark photoperiod and 80–85% relative humidity at 26 ± 2°C. The quartz sand was preprepared by steam sterilization for 3 h. After 30 days of cultivation, the plants were washed three times with sterile double-distilled water and then transplanted into 50-mL sterilized flasks containing 20 mL of sterile distilled water. The plants were infected with bacterial suspension at a final concentration of 2 × 10^6^ CFU/mL (OD_600_ = 1.0; 1 × 10^9^ CFU/mL in sterile distilled water) in the plant growth solution. Each treatment had three replicates, and each replicate contained a mixture of five plants. After 72 h, the liquid medium was collected and filtered through a 0.22-μm sterile filter (Millipore) to remove the pathogen and residues. Filtered fluids were then lyophilized and dissolved in 5 mL of methanol and stored at −20°C for subsequent high performance liquid chromatography (HPLC) analysis.

### Identification of Phenolic Acids

High-performance liquid chromatography (HPLC, Agilent 1260 series, Agilent Technologies, Santa Clara, CA, United States) was used to separate and identify the phenolic acids in tobacco root exudates as described previously with minor modifications (Hao et al., 2010). The HPLC analysis was performed on an Agilent SB-Aq column (5 μm, 250 × 4.6 mm; Agilent, United States) at 40°C. Methyl alcohol (solution A) (Fisher Co.) and 2% (v/v) acetic acid (pH = 2.59) (solution B) were used as the mobile phases. A total of 10 μL of sample was eluted for 35 min with a flow rate of 0.8 mL/min and detected at 280 nm with a UV detector. The gradient profile was as follows: for 0 min, 100% solution B and 0% solution A; for 21.5 min, 50% solution B and 50% solution A; for 23.5 min, 60% solution B and 40% solution A; for 27 min, 35% solution B and 65% solution A, for 30 min, 0% solution B and 100% solution A. The standard phenolic acids used in this study included caffeic acid, *p*-coumaric acid, cinnamic acid, ferulic acid, vanillic acid, and benzoic acid. Peaks were determined by comparing the retention time with standards. The concentration of each compound in tobacco root exudates was obtained from the peak areas using an external standard.

### Determination of Antibacterial Activity

The minimum inhibitory concentration (MIC) and the minimum bactericidal concentration (MBC) of caffeic acid (CA) against *R. solanacearum* were determined by an agar dilution assay at different concentrations (75, 100, 150, 200, 250, and 500 μg/mL) in Petri dishes. One hundred microliters of overnight-cultured *R. solanacearum* suspension diluted to 1 × 10^5^ CFU/mL was coated directly onto each antibiotic-containing agar dilution plate. Dimethyl sulfoxide (DMSO) at 0.5% was used as the control treatment, and this concentration was used in all the following tests. The plates were incubated at 30 ± 1°C. The minimum inhibitory concentration (MIC) was the lowest concentration at which plates had no colony formation or fewer than three colonies after culturing for 48 h. The minimum bactericidal concentration (MBC) was determined as the lowest concentration after culturing for 96 h.

### Cell Viability Measurement

According to a previous study (Cai et al., 2018), the plate counting method was adopted to determine bacteria viability. Overall, 100 μL of fresh *R. solanacearum* cells (1 × 10^5^ CFU/mL) were directly coated onto NB medium plates containing different concentrations of caffeic acid (75, 100, 150, and 200 μg/mL). After being cultured for 2 days in an incubator, the CFU was counted on the agar plates. Each experiment was repeated four times. The cell viability was calculated as the following formula:

$$\mathrm{Cell}\mathrm{viability}\left(\%\right)=\frac{\text{V}\,1}{\text{V}}\times 100\%$$

where V and V_1_ represent the number of colony-forming units on the control plates and caffeic acid-treated plate.

### Growth Curves Assay

The antimicrobial activity of caffeic acid was evaluated by examining the OD growth curves. Caffeic acid was added into 100 mL of NB medium broth to obtain a final concentration of 75, 100, 150, or 200 μg/mL, and the control culture was supplemented with sterile water. The medium was inoculated with 100 μL of a freshly cultured suspension of *R. solanacearum* (1 × 10^9^ CFU/mL). Then, the cultures were incubated at 180 r/min for 24 h at 30°C, and cell growth was monitored spectrophotometrically (the optical density at 600 nm was recorded at 2 h intervals). All treatments were repeated three times and the average value was calculated.

### Morphology Observation of *R. solanacearum*

The morphology of *R. solanacearum* cells in the presence of caffeic acid was evaluated by using scanning electron microscopy. First, *R. solanacearum* in the logarithmic growth phase was diluted into a 10^8^ CFU/mL suspension. Then, caffeic acid was added to the bacterial suspension to reach a final concentration of 200 μg/mL. After incubation with shaking at 30°C for 12 h, the cells were collected and washed three times with 0.1 mol/L pH 7.0 phosphate buffer and then fixed in a 2.5% glutaraldehyde solution at 4°C overnight. Scanning electron microscopy was performed according to Li et al. (2016).

### LIVE/DEAD Assays to Infer Cell Membrane Damage

A LIVE/DEAD BacLight Bacterial Viability Kit (Molecular Probes, Eugene, OR, United States) was used as the identification tool to verify the membrane damage of bacterial cells, as previously described (Cai et al., 2018). First, *R. solanacearum* was cultivated in NB medium at 30°C for 10 h without shaking. Then, the supernatant was removed by centrifugation at 6000 × *g* and fresh NB medium blended with caffeic acid (150 and 200 μg/mL) was added to the culture. After static incubation for 4 h, 10 μL of reagent mixture (SYTO 9 and propidium iodide) was added to the bacterial suspension (washed in sterile water) and stained in the dark for 15 min. Next, the bacteria were observed by an inverted fluorescence microscope (Axio Observer A1, Germany) at a 485/530 nm wavelength. The percentage of live cells was calculated according to the following equation:

$$\begin{array}{c} \mathrm{The}\,\mathrm{percentage}\,\mathrm{of}\\ \mathrm{live}\mathrm{cells}\left(\%\right) \end{array}=\frac{{\text{F}}_{\mathrm{cell},\mathrm{green}}-{\text{F}}_{\mathrm{cell},\mathrm{red}}}{{\text{F}}_{\mathrm{cell},\mathrm{green}}}\times 100\%$$

where F*_*cell,green*_* = the number of bacterial cells strained by SYTO 9; F*_*cell,red*_* = the number of bacterial cells strained by propidium iodide.

### Determination of Biofilm Formation

A biofilm formation assay was performed by employing the crystal violet assay (Peeters et al., 2008). A 5-mL centrifuge tube contained 3 mL of NB medium and CA with final concentrations of 75, 100, 150, or 200 μg/mL. Overnight-cultured *R. solanacearum* suspension (OD_600_ = 1.0) was added to the centrifuge tube at a proportion of 2:1000, and 200 μL of the mixed suspension was added to each well of a 96-well culture plate. Each treatment had 12 replicates. After incubation without shaking at 30°C for 24 h, the medium was removed, and the wells were washed twice with distilled water. Then, 220 μL of crystal violet was added to the wells for dyeing for 40 min. The unbound crystal violet stain was gently removed, and the wells were washed twice. The bound crystal violet was dissolved by adding 220 μL of 95% ethanol, and the plate was then incubated at room temperature for 30 min. The biofilm formation value was measured at 490 nm by a Thermo Scientific Multiskan GO analysis system. All assays were conducted with three biological replicates.

### Analysis of the Expression of Biofilm Formation-Related Genes

Gene expression was analyzed by an RT-PCR assay. Briefly, 1 mL of NB medium with a final CA concentration of 75, 100, 150, or 200 μg/mL was added to each well of a 48-well culture plate. Ten milliliters of overnight-cultured *R. solanacearum* suspension (OD_600_ = 1.0) was added to the plates. The plates were incubated at 30 ± 1°C for 24 h. Each treatment had three replicates, and each replicate included four wells. Cells were harvested, and total RNA was extracted using TRIzol reagent (Tiangen Biotech Co., Ltd., Beijing, China). The cDNA was synthesized using an iScript cDNA Synthesis Kit (Bio-Rad, Hercules, CA, United States). The primers for the virulence-related genes are listed in Table 1, and *SerC* was used as the reference gene. The relative expression of genes was measured according to the methods previously described by Li et al. (2017b). All assays were conducted with three biological replicates.

**TABLE 1:** DNA primers used in this study.

Primers	Characters or sequences (5′–3′)	References or sources
*SerC-*F	CCCACCTACGCCATCTATGT	Yang et al., 2018
*SerC-*R	TTGAGGAAGAACGGCACATT	
*lecM-*F	TCAGCCCAGCGGCCAGTTCAG	Mori et al., 2016
*lecM-*R	GGCTCAGCAAGGTGTATTCACGC	
*xpsR-*F	AGATCGACATAGCGCTGCTT	Yang et al., 2017
*xpsR-*R	TTACTTTGCGGACCTGCTCT	
*epsE-*F	CTGGATAAAGCCACGCAAAG	
*epsE-*R	CAGTGGTACATCGCCATCAC	

### Determination of Enzyme Activity, Hydroxyproline, and Lignin Content

The effect of CA on the activity of defensive enzymes associated with phenylpropanoid metabolism was assessed under hydroponic conditions. Tobacco seedlings with four or five true leaves were chosen and cultivated in a flask hydroponic system inoculated with 1/2 Hoagland’s solution. One group was treated with 1 mL of CA (200 μg/mL), one group was treated with 1 mL of *R. solanacearum* (OD_600_ = 0.1, 1 × 10^8^ CFU/mL), and the control group was treated with distilled water. Each treatment was completed in three replicates with 60 plants. After treatment, healthy tobacco plants in each treatment were collected at 1, 3, 6, 10, and 15 days. The roots and stems of each plant were rapidly frozen in liquid nitrogen, ground into powder, and stored at −80°C for subsequent determination. The activity of phenylalanine ammonia-lyase (PAL) and peroxidase (POD) was assayed according to Zhang et al. (2020). The PAL and POD activities were represented as U min^–1^g^–1^ FW. In addition, the content of lignin and hydroxyproline was determined and analyzed by the method of Yuan et al. (2013) and Basavaraju et al. (2009).

### Determination of Colonization of *R. solanacearum* in Tobacco Roots

Colonization of *R. solanacearum* was completed in tobacco roots grown under hydroponics. Briefly, tobacco seedlings with four to five leaves were washed three times with distilled water, and tobacco roots were dipped in a solution of caffeic acid for 30 min. The treated tobacco seedlings were cultivated in a flask hydroponic system inoculated with a bacterial suspension at a final concentration of 1 × 10^7^ in 1/4 Murashige and Skoog (MS) solution. After 3 days of incubation at 28°C in a growth chamber set to a 14 h light/10 h dark photoperiod with 80–85% relative humidity, the population of *R. solanacearum* adhering to the tobacco root was detected. Each treated 0.1 g root sample was ground in 0.9 mL of sterile water using a mortar until finely homogenized. Then, the number of *R. solanacearum* in the suspension was determined on NA medium for 48 h at 30°C. Each treatment included four replicates, and all assays were conducted with three biological replications.

### Indoor Pot and Field Experiments

To evaluate the control of CA in tobacco bacterial wilt control, an indoor pot experiment was performed in the greenhouse. First, tobacco seedlings were cultivated in a matrix at 28°C and 60% relative humidity. After 3 weeks, the tobacco seedlings were transplanted into pots and grew to four to five true leaves were reached. Then, 5 mL of CA at concentrations of 100, 150, and 200 μg/mL was applied to the tobacco roots, and the negative control was treated with distilled water. Five milliliters of 1 × 10^8^ CFU/mL bacterial suspension was inoculated on each plant root at a distance of 4 cm from the stem base after 3 days. Each treatment had 30 plants and was replicated three times. A total of 120 plants were used to evaluate the incidence of bacterial wilt disease. Disease progress was scored every 2 days on a disease index scale from 0 to 4, as previously described (Li et al., 2017b).

The field experiment was performed in Pengshui, Chongqing, China. In this experimental field, tobacco was planted every year, and serious bacterial wilt occurred. After flue-cured tobacco was transplanted for 40 days, 20 mL of CA at concentrations of 100, 150, and 200 μg/mL was poured into the stem base of the tobacco plant, and the negative control was treated with distilled water. Each treatment plot was randomly designed with an area of approximately 100 m^2^ (length × width = 10 m × 10 m), and 100 tobacco plants were distributed in each plot. Each treatment had three plots. After treatment, disease progress was scored every 10 days on a disease index scale from 0 to 9 (Li et al., 2017a). The disease index (DI) and control efficiency (CE) were calculated using the following formulas:

$$\text{DI}=\frac{\sum \left(n_{i}\times v_{i}\right)}{N\times 9}\times 100$$

where *n*_*i*_ is the number of plants with disease index *v*_*i*_ (0, 1, 3, 5, 7, or 9), and *N* is the total number of plants used in each treatment.

$$\text{CE}=\frac{D\,I\,C-D\,I\,T}{D\,I\,T}\times 100\left(\%\right)$$

where *DIC* represents the disease index in the control, and *DIT* represents the disease index in the treatment.

## Data Analysis

The data among the treatments were compared and statistically analyzed using one-way analysis of variance (ANOVA), Duncan’s multiple range tests with a *p* value of 0.05, and independent-sample *t*-tests with *p* values of 0.05 and 0.01. SPSS version 17.0 was used for the statistical analyses.

## Results

### Enrichment of Caffeic Acid Enrichment in Tobacco Root Exudates After *R. solanacearum* Infection

**FIGURE 1:**
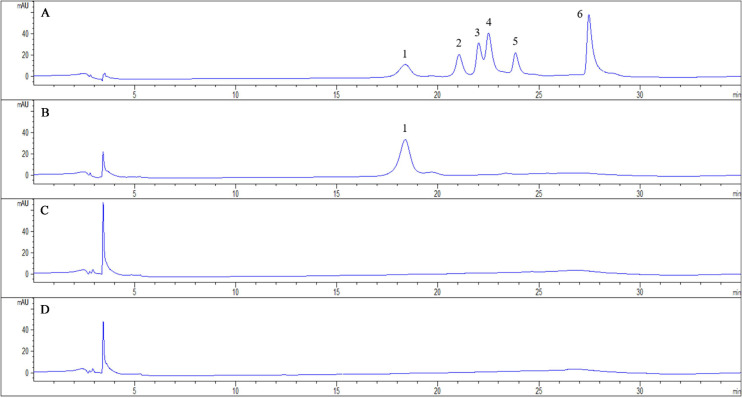
Chromatogram of exudates from tobacco roots and *R. solanacearum* and their interaction. The chromatograms are of phenolic acids detected with HPLC both in standard chemicals and root exudates collected 3 days post-inoculation from the interaction of tobacco and *R. solanacearum*. The peaks in **(A)** represent the following standard compounds: 1, caffeic acid; 2, syringic acid; 3, benzoic acid; 4, *p*-coumaric acid; 5, ferulic acid; and 6, cinnamic acid. **(B–D)** represent the chromatograms of the interaction of tobacco and *R. solanacearum*, *R. solanacearum* only, and tobacco only, respectively.

We measured the content of induced phenolic acids in the root exudates of pathogen-infected tobacco by using HPLC. And root exudates of tobacco infected with *R. solanacearum* were predominantly compared with solvent extracts of tobacco-only and pathogen-only. The tobacco plants with *R. solanacearum* infection showed no symptoms of disease during the 3-day sampling period. After comparison with the standard phenolic acids HPLC chromatogram (Figure 1A), the chromatograph chart revealed that caffeic acid was detected and increased in the profiles of root exudates under pathogen infection (Figure 1B). However, caffeic acid was never detected in pathogen-only samples (Figure 1C), or tobacco-only samples (Figure 1D), which suggested that caffeic acid was induced by tobacco under *R. solanacearum* infection. Additionally, the concentration of caffeic acid was 1.95 ± 0.29 μg/mL in root exudates, based on comparing the peak of the peaks of the standard.

### Antibacterial Activity of Caffeic Acid Against *R. solanacearum in vitro*

**FIGURE 2:**
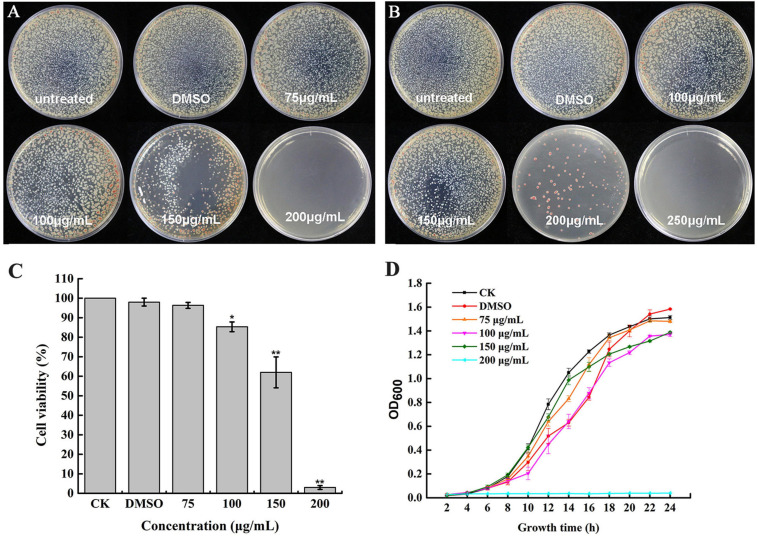
Antibacterial activity of caffeic acid against *R. solanacearum*. The growth of *R. solanacearum* on plates was determined at 48 **(A)** and 96 h **(B)**. The *R. solanacearum* cell viability rate **(C)** and growth curve after exposure to different concentrations of caffeic acid **(D)**. The assay was independently replicated four times. The error bars indicate the standard errors from four independent replicates, and * and ** indicate significant differences identified according to independent-sample *t*-tests with a *p*-value of 0.05 and 0.01, respectively.

To determine whether caffeic acid could inhibit the growth of *R. solanacearum*, the MIC and MBC of caffeic acid against *R. solanacearum* were measured using an agar dilution assay. The MIC of caffeic acid was 200 μg/mL (Figure 2A). As shown in Figure 2B, the MBC of caffeic acid against *R. solanacearum* was 250 μg/mL. The growth of bacteria on plates under DMSO treatment at 48 and 96 h was not affected. In addition, bacteria viability was determined by using the plate counting method. As shown in Figure 2C, the survival rate at a 200 μg/mL concentration remarkably only reached 3.32% compared with untreated which almost completely induced death. Furthermore, 200 μg/mL of caffeic acid significantly inhibited the growth of *R. solanacearum* in NB medium, whereas less inhibition was found for a 75–150 μg/mL concentration of caffeic acid (Figure 2D). At a 200 μg/mL concentration, the growth cure results demonstrated that the bacteria were almost killed.

### Caffeic Acid Destroys Morphological Structure of *R. solanacearum*

**FIGURE 3:**
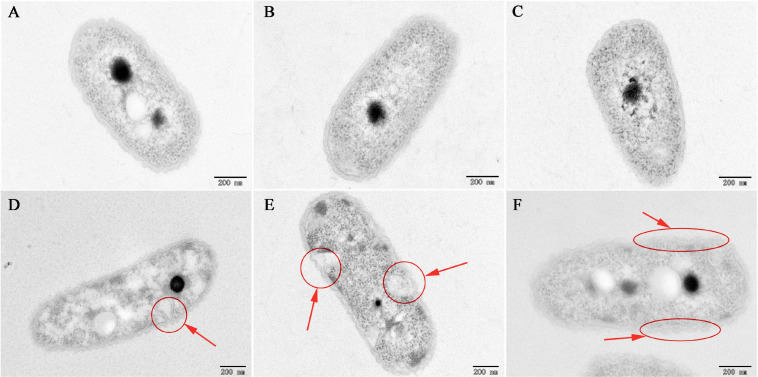
TEM images of *R. solanacearum* cells treated with CA at a 200 μg/mL concentration. **(A–C)** Untreated cells. **(D–F)** Cells treated with a 200 μg/mL concentration of CA. Cells were incubated for 12 h with shaking at 180 rpm at 30°C. The red circle and arrows represent the damaged bacterial membranes and irregular cavities in cells **(D,E)**. The red ellipse and arrows represent the thinning and irregularity of the cell membrane in cells **(F)**.

To investigate the mechanism of the antibacterial activity of caffeic acid, the cell morphology of *R. solanacearum* treated with 200 μg/mL of caffeic acid was monitored using TEM. Under our experimental conditions, the untreated bacteria presented typical *R. solanacearum* morphology with intact membrane structure integrity (Figures 3A–C). In contrast, caffeic acid treatment obviously destroyed the membrane structure of the bacterial cells, resulting in irregular hollowness and cell membrane thinning (Figures 3D–F). These results were consistent with the observed antibacterial activity of caffeic acid through damaging the cell wall of *R. solanacearum*.

**FIGURE 4:**
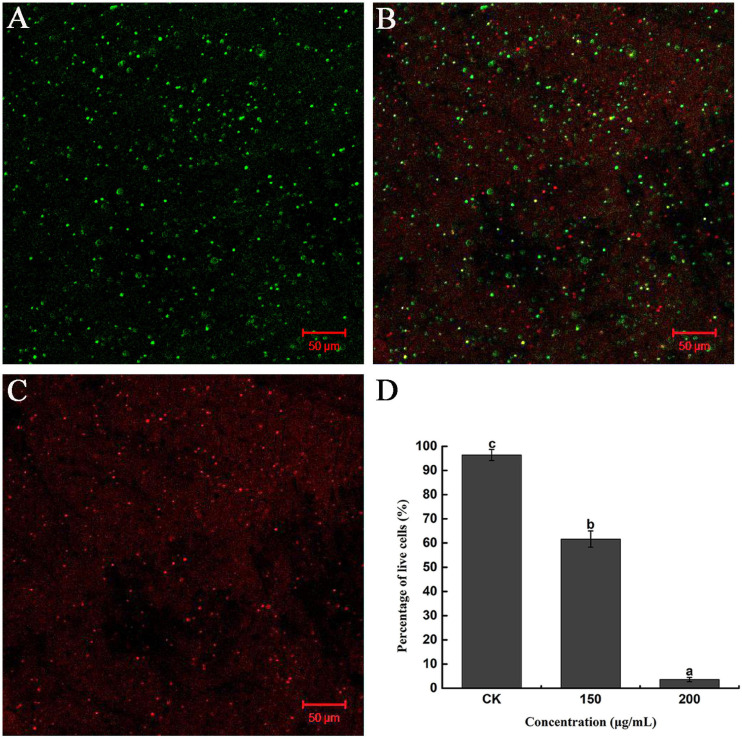
Fluorescence microscope image of *R. solanacearum* cells treated with caffeic acid. **(A–C)** Confocal fluorescence microscopic images were obtained using the LIVE/DEAD BacLight Bacterial Viability and Counting Kit and treatment with caffeic acid at 0, 150, and 200 μg/mL concentrations, respectively. **(D)** The percentage of live cells supplemented with caffeic acid after incubation for 10 h. Error bars indicate the standard deviation and different letters indicate significant differences between caffeic acid treatment and control (*P* < 0.05, Duncan’s test).

Furthermore, the severity of membrane destruction was determined by quantification analysis using a LIVE/DEAD BacLight Bacterial Viability Kit. After staining with the LIVE/DEAD Kit, cells with damaged membranes were considered to be dead and stained red, whereas cells with an intact membrane were stained green. In the control image, 96.36% of *R. solanacearum* cells were stained green, indicating that a high percentage of live cells were incubated after 10 h of incubation (Figures 4A,D). However, after incubation with 200 mg/mL of caffeic acid, there were a large number of dead cells (Figure 4C), and the percentage of live cells was only 3.62%, which was significantly lower than that of the control (Figure 4D). Collectively, caffeic acid damaged the membrane structure of *R. solanacearum* cells, possibly resulting in cell injury or death.

### Evaluation of *R. solanacearum* Biofilm Formation in Response to Caffeic Acid

**FIGURE 5:**
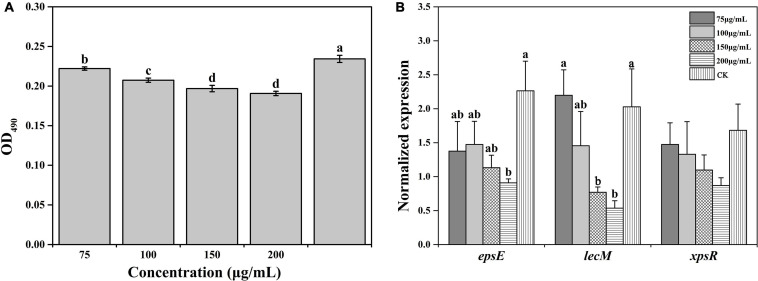
Effect of caffeic acid on biofilm formation **(A)** and the expression of related genes **(B)** of *R. solanacearum* at different concentrations ranging from 75 to 200 μg/mL. This assay was independently replicated three times, and the OD_490_ value was determined at 24 h. *SerC* was used as the reference gene to normalize the gene expression using the 2^–△△Cq^ method. The error bars indicate the standard errors from three independent replicates, and the lowercase letters indicate significant differences identified according to Duncan’s test (*p* < 0.05).

Biofilm formation contributes to resisting adverse external environments and the erosive effects of harmful substances, maintaining the relative stability of the living environment. The biofilm formation under all concentrations of caffeic acid treatment was lower than the biofilm formation of the control. The concentration of caffeic acid determined the inhibition of *R. solanacearum* biofilm formation (Figure 5A). Compared with biofilm formation in the control, biofilm formation after 75–200 μg/mL caffeic acid treatments was significantly reduced by 5.22, 11.48, 15.98, and 18.62% at 24 h.

Furthermore, we evaluated the effect of caffeic acid on the expression of genes related to the biofilm formation of *R. solanacearum*. The results showed that the expression of genes was downregulated to varying degrees under caffeic acid treatment (Figure 5B). Among these genes, in terms of the induction of *epsE* expression, 75–200 μg/mL of caffeic acid inhibited the expression of *epsE*, which was downregulated by 1. 65-, 1. 54-, 2. 0-, and 2.49-fold compared with the expression of *epsE* in the control. Additionally, 200 μg/mL of caffeic acid showed a significant difference at the *p* < 0.05 level. Analysis of the expression level of the *lecM* gene showed that caffeic acid at concentrations of 100–200 μg/mL inhibited the expression of *lecM* with decreases of 1. 39-, 2. 63-, and 3.78-fold compared with the expression of *lecM* of the control. The expression of *xpsR* was inhibited by the four concentrations of caffeic acid. However, no significant differences were detected among the five treatments. These results indicated that the antibiofilm effect of caffeic acid might be related to the repression of the related genes *lecM* and *epsE*.

### Caffeic Acid Increases Tobacco Plant Resistance

**FIGURE 6:**
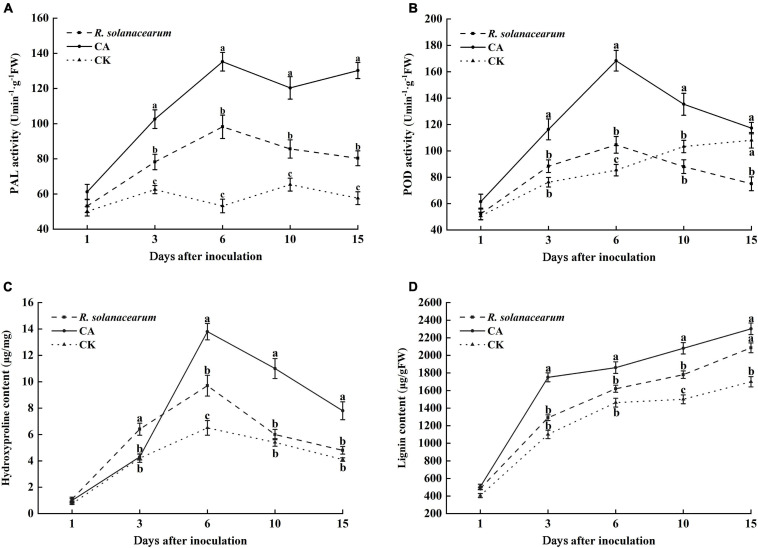
Effect of caffeic acid on PAL **(A)**, POD **(B)**, hydroxyproline **(C)**, and lignin **(D)** activity from tobacco. CA, *R. solanacearum*, and CK represent caffeic acid concentrations of 200 μg/mL, 1 mL *of R. solanacearum* (1 × 10^8^ CFU/mL), and the control (sterilized distilled water), respectively. The mean value in both directions was calculated from triplicate plates. The error bars indicate the standard errors from three independent replicates, and the lowercase letters indicate significant differences identified according to Duncan’s test (*p* < 0.05).

PAL is an important disease-resistant enzyme in the phenylpropanol metabolism pathway. Thus, the PAL activity of tobacco under the influence of CA and *R. solanacearum* was determined. The results showed that the PAL activity of the CA-treated groups was significantly higher than that of *R. solanacearum* and CK groups throughout the inoculation time (Figure 6A). 3 days after inoculation, the PAL activity of tobacco was increased in all treatments. However, the PAL activity of *R. solanacearum*-treated and CK plants decreased from 10 to 15 days. CA treatment reached its maximum at 6 and 15 days, at which time the activity was approximately 37, 50 and 82, 73 enzyme units higher than that of *R. solanacearum* and CK, respectively.

POD plays a key role in the final synthesis of plant lignin, which is an important indicator of plant resistance. The results showed that the change trend of POD activity in the CA- and *R. solanacearum*-treated groups was similar, and enzyme activity first increased at 6 days and then decreased (Figure 6B). However, the POD activity of the CA-treated group remained significantly higher than that of the *R. solanacearum*-treated group after inoculation for 3 days, showing enhancements of 28, 64, 47, and 42 units, respectively. In addition, the POD activity of CK gradually increased and was significantly higher than the POD activity of the *R. solanacearum*-treated groups at 15 days. Furthermore, the content in the CA-treated groups was significantly higher than that of *R. solanacearum* and CK (*P* < 0.05), showing enhancements of 16.85–35.65 and 48.57–59.09% between 3 and 10 days (Figure 6D). The lignin content during the whole inoculation period was CA > *R. solanacearum* > CK.

Likewise, CA and *R. solanacearum* treatment increased hydroxyproline content in the tobacco with the treatment effect CA > *R. solanacearum* > CK (Figure 6C). After inoculation, the content of hydroxyproline in all groups increased rapidly and reached a maximum on the 6th day, at which time the content of CA-treated groups was 4.1 and 7.3 μg/mg higher than that of *R. solanacearum* and CK, respectively (Figure 6C). Moreover, the changes in the content of treated *R. solanacearum* were similar to the changes in the content of CK, and the maximum value was 9.7 μg/mg higher than the 6.5 μg/mg of CK. Thus, CA treatment could promote the accumulation of hydroxyproline.

### Caffeic Acid Decreases the Root Colonization of *R. solanacearum*

**FIGURE 7:**
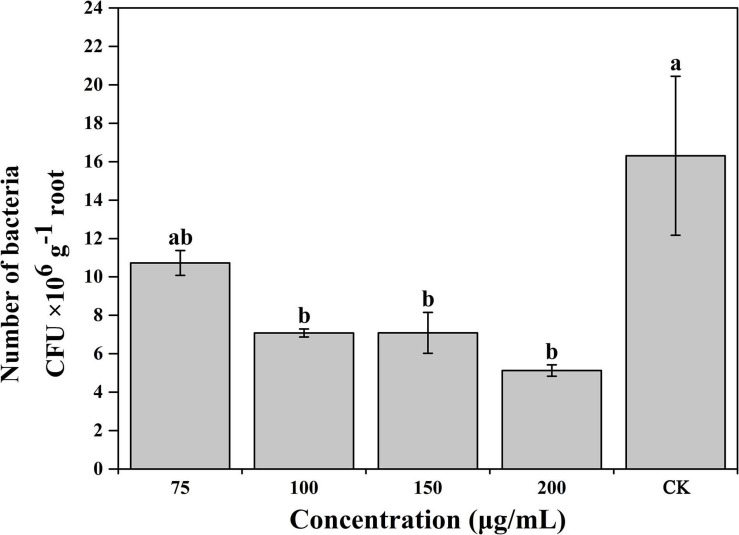
Influence of caffeic acid on *R. solanacearum* colonization of tobacco roots. The error bars indicate the standard errors from three independent replicates, and the lowercase letters indicate significant differences identified according to Duncan’s test (*p* < 0.05).

Based on the results of the experiments described above, caffeic acid had a negative effect on the virulence of *R. solanacearum*. Thus, we subsequently evaluated the effect of caffeic acid on the colonization of *R. solanacearum* in tobacco roots under hydroponic conditions. Caffeic acid significantly decreased the population of *R. solanacearum* that colonized the roots of tobacco plants at concentrations of 100–200 μg/mL (Figure 7). Caffeic acid significantly reduced the colonization of *R. solanacearum* at concentrations from 100 to 200 μg/mL, which was 2. 3-, 2. 3-, and 3.18-fold lower than the control.

### Application of Caffeic Acid Reduces the Occurrence of Bacterial Wilt

**FIGURE 8:**
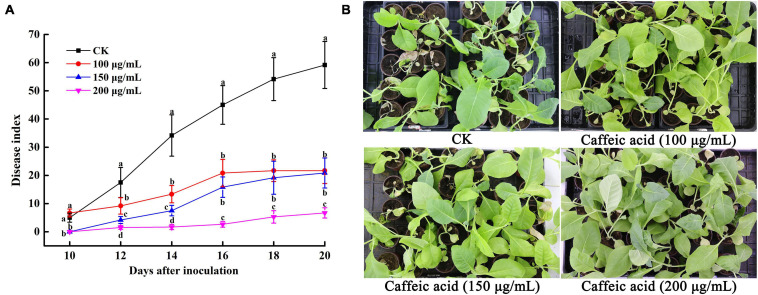
Disease index of tobacco bacterial wilt CA treatment **(A)**. Disease symptoms of representative tobacco seedlings inoculated with *R. solanacearum* for 20 days **(B)**. Unwounded tobacco plants were soil-soak inoculated at concentrations of 5 mL (1 × 10^8^ CFU/mL) and inoculated at 28 ± 1°C after treatment with 100, 150, 200 μg/mL PCA. Untreated plants were treated with sterile water. The bars indicate the standard error of the mean of three replicates. The lowercase letters indicate significant differences identified according to Duncan’s test (*p* < 0.05).

The results of the experiments described above suggested that CA could inhibit *R. solanacearum* and induce plant resistance. Thus, we determined the effects of caffeic acid on tobacco bacterial wilt through a pot experiment. The results showed that caffeic acid treatment reduced and delayed the incidence of tobacco bacterial wilt, and the incidence peak was significantly lower than that of the control (Figure 8A). 10 days after inoculation, the control and caffeic acid-treated plants developed symptoms of bacterial wilt. Then, the incidence rate of control treatment increased rapidly, and the disease index was higher than the disease index of caffeic acid treatment. Meanwhile, 200 μg/mL of caffeic acid could significantly reduce the disease index compared with 100 and 150 μg/mL treatments from 12 to 20 days. Moreover, 20 days after inoculation, the disease index of tobacco bacterial wilt of 200 μg/mL of caffeic acid was 6.67, which was significantly lower than the disease index of the control, 100 and 150 μg/mL at 59.17, 21.67, and 20.83, respectively (Figure 8).

**FIGURE 9:**
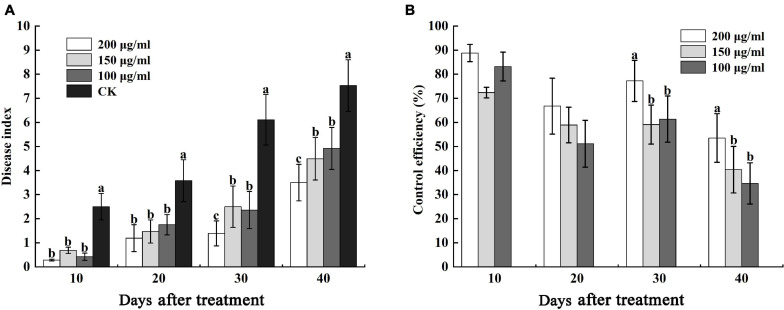
Effects of caffeic acid at concentrations of 100–200 μg/mL on the disease index and control efficiency of tobacco bacterial wilt. **(A)** The disease index of tobacco plants infected with *R. solanacearum* was determined. **(B)** The control efficiency of caffeic acid toward tobacco bacterial wilt. The lowercase letters indicate significant differences among the different treatments (Duncan’s test, *p* < 0.05).

Furthermore, we determined the control efficiency of CA on bacterial wilt in tobacco fields. At 10 and 20 days after treatment, the disease indexes of CK were 2.50 and 3.58, respectively, which were higher than different concentrations of CA, but there was no obvious difference in 100 μg/mL, 150 μg/mL, and 200 μg/mL CA (Figure 9A). The control efficiencies of 100–200 μg/mL CA and CK were 88.80, 72.40, and 83.20% and 66.76, 58.94, and 51.12%, respectively (Figure 9B). 30 and 40 days after treatment, various concentrations of CA application exerted marked effects on bacterial wilt, and the disease index reduced at concentrations ranging from 100 to 200 μg/mL reaching nearly 4.72, 3.61, 3.75 and 4.03, 2.64, 3.61 lower values compared with the values of the control, respectively. Moreover, the control efficiencies of 200 μg/mL CA application, 77.25 and 53.52%, were significantly higher than the control efficiencies of other concentrations.

## Discussion

Root exudates are an important medium for the exchange of matter and energy between plants and the external environment and regulate the interactions between plants and microorganisms (Sasse et al., 2017). More reports illustrate that changes in the variety and in the quantity of plant root exudates are a direct response to pathogen attack. In rhizobia-inoculated rice plants, the synthesis of phenolic compounds increases compared with uninoculated plants. The phenolic compounds mediate induced system resistance and protect plants from pathogenic stresses (Mishra et al., 2006). Soybean hairy roots produce isoflavonoids in response to *Fusarium solani* infection, and the isoflavonoids confer resistance against this fungus (Lozovaya et al., 2004). Our results demonstrated that caffeic acid in root exudates increased significantly in response to *R. solanacearum* attack compared with the absence of the pathogen under hydroponic conditions (Figure 1). Increasing the concentration of caffeic acid activates the phenylpropanoid metabolism pathway, which is also stimulated by fungal pathogens and environmental stress (Sarma and Singh, 2003). Furthermore, *in vitro*, caffeic acid showed antibacterial activity against *R. solanacearum* by inducing irreversible damage to the cell membrane (Figures 3, 4) and activating plant defenses by increasing the production of phenylpropanoids (Figure 6), and reduced the incidence of tobacco bacterial wilt in the pot and field experiments (Figures 8, 9). These experiments demonstrated that plants develop defense responses by secreting antimicrobial compounds in response to the infection of a pathogen that changes the root exudate patterns and inhibits the invasion of the pathogen.

By definition, inducible antimicrobial compounds that are synthesized and accumulated after exposure to microorganisms, and which cannot be detected in healthy plants are called phytoalexins (Vanetten et al., 1994). For instance, rice challenged by the blast fungus shows targeted accumulation of momilactone A, and the secretion levels of momilactone increased 500- to 1000-fold, which also exhibited antifungal activity at a concentration of 0.3 mM (Hasegawa et al., 2010). During barley root infection by *Fusarium*, the predominant exudates’ *t*-cinnamic acid was up to 0.5 μM, which was 45-fold higher than the hydroponic cultures without *F. graminearum*. Moreover, bioassays indicated that 10 μM of *t*-cinnamic acid was the minimum concentration for inhibition of spore germination (Lanoue et al., 2010a). Similarly, upon *Pseudomonas aeruginosa* infection, rosmarinic acid was largely excreted in sweet basil root exudates, and the maximum concentration (14–15 μg/mL) occurred 6 days post-infection. However, rosmarinic acid was not detected in control plants without *P. aeruginosa* infection (Walker et al., 2004). In this study, our results indicated that caffeic acid in response to *R. solanacearum* attack was secreted, whereas caffeic acid was not observed in healthy tobacco (Figure 1). We showed a maximum root secretion of caffeic acid at 1.95 ± 0.29 μg/mL, whereas the minimum concentration for inhibited *R. solanacearum* was 200 μg/mL. This gap raises the question of the effective concentration of exudates in the rhizosphere. In this study, only 20 mL of hydroponic solution were collected and extracted. However, a much higher local concentration was distributed in the narrow zone around roots. And the real concentrations of exudates in the rhizosphere were likely underestimated. To date, hydroponics and soil solutions cannot accurately detect the concentration of root exudates (Eva and Jones, 2018). Thus, new sampling collection methods such rhizobox and soil exudation traps are being developed to overcome this problem (Oburger et al., 2013; Eva and Jones, 2018).

Caffeic acid is an important natural product with a variety of biological activities, such as antibacterial, antiviral, and anti-inflammatory activity (Tyszkaczochara et al., 2017; Wu et al., 2017). In particular, our results proved that caffeic acid was an effective antibacterial compound against *R. solanacearum* (Figure 2). According to some research, the cell membrane is the primary target for the antimicrobial activity of most phytochemicals (Chen et al., 2016). Recently, coumarins, protocatechualdehyde, and resveratrol inhibited *R. solanacearum* by damaging bacterial cell membranes (Chen et al., 2016; Li et al., 2016; Yang et al., 2016). Based on the fluorescence microscopy and scanning electron microscopy results, caffeic acid might destroy the cell membrane, causing the death of *R. solanacearum* (Figures 3, 4). Besides, biofilms can provide protection against plant defense responses, thereby promoting colonization and accelerating disease. In the case of pathogens, vascular pathogens that cause blockage and tissue damage largely depend on biofilm (Danhorn and Fuqua, 2007). In this case, plants produce a battery of compounds in response to pathogens through inhibiting biofilm formation on plant roots (Dixon, 2001). For instance, protocatechualdehyde and gallic acid showed a significant antipathogenic effect against *R. solanacearum* by suppressing virulence factors, such as inhibiting biofilm formation and reducing adhesion to the host (Farag et al., 2015; Li et al., 2016). In addition, salicylic acid acts to directly suppress virulence genes of *exoT*, *exsB*, and *exsC* in *P. aeruginosa*, inhibiting biofilm formation on *Arabidopsis thaliana* roots (Prithiviraj et al., 2005). Yang et al. (2017) reported that umbelliferone could significantly decrease gene expression of the type III secretion system (T3SS) and biofilm formation, and reduced colonization of *R. solanacearum* in tobacco roots. In particular, *lecM* and *epsE* in T3SS are important genes involved and regulated in biofilm formation. And the gene deletion of *lecM* caused a significant decrease in biofilm formation of *R. solanacearum*, leading to a loss of virulence on tomato plants (Mori et al., 2016). In this study, the results found that caffeic acid repressed the gene expression of *lecM* and *epsE*, and decreased the biofilm formation and colonization ability of *R. solanacearum* (Figures 5, 7).

Elicitors can induce a line of defense responses in plants and enhance plant disease resistance by activating the activity of resistance-related enzymes and stimulating the biosynthesis of secondary metabolites, antitoxins, and lignin (Jian et al., 2005). Exogenous application of elicitors such as salicylic acid, jasmonic acid, and methyl jasmonate could significantly increase the activities of PAL and POD enzymes and reduce the disease incidence of tomato bacterial wilt (Mandal et al., 2013). PAL and POD are involved in the oxidation of polyphenols and the biosynthesis of phenols and plant lignin, which contributes to enhancing plant resistance to disease (Gorny et al., 2002; Hu et al., 2014). In this report, exogenous caffeic acid markedly increased PAL and POD enzymatic activities, which were significantly higher than those in the control group (Figures 6A,B). Similarly, Zhang et al. (2020) found that caffeic acid treatment increased POD and PAL enzyme activity in apples and significantly inhibited *Botrytis cinerea* infection. Additionally, lignin and hydroxyproline are major secondary products in plants with important disease resistance functions. Liu et al. (2010) indicated that riboflavin treatment could promote the accumulation of scopoletin and lignin by 48.0% compared with *R. solanacearum* challenge to protect tobacco against *R. solanacearum*. In the present study, the results also showed that the accumulation of lignin and hydroxyproline in tobacco induced by caffeic acid was significantly higher than the accumulation of lignin and hydroxyproline in *R. solanacearum* (Figures 5C,D). Moreover, Bubna et al. (2011) also found that caffeic acid effectively promoted lignin accumulation to strengthen the cell wall, thus reducing the invasion of gray mold. Therefore, in addition to antibacterial function, caffeic acid treatment effectively activated PAL and POD and promoted lignin and hydroxyproline accumulation, thereby forming a stronger physical barrier against the invasion of *R. solanacearum*.

The use of natural compounds is a potent way to control phytopathogenic bacteria (Jiménez-Reyes et al., 2018). For instance, irrigating roots with hydroxycoumarins, daphnetin and protocatechualdehyde, significantly delayed the occurrence of tobacco bacterial wilt, with the control efficiency of 70–80% (Li et al., 2016; Yang et al., 2016).

In pot experiments, the application of lysine to sand culture medium and soil could reduce tomato bacterial wilt by 58–100% (Posas et al., 2007). Tannins isolated from *Sapium baccatum* extract exhibited strong antibacterial activity against *R. solanacearum*, and the crude extract of *S. baccatum* also reduced the development of tomato bacterial wilt by 63–83% under greenhouse conditions after 14 days of infection (Vu et al., 2017). Yang et al. (2016) demonstrated that irrigating roots with hydroxycoumarins 24 h before inoculation with *R. solanacearum* significantly delayed the occurrence of tobacco bacterial wilt, with a control efficiency of 38.27–80.03% at 6–16 days after inoculation. In this study, we also found that caffeic acid could effectively control tobacco bacterial wilt, with control efficiencies of 33.33–48.27 and 51.12–88.80% in greenhouse and field experiments, respectively (Figures 8, 9). The results suggested that caffeic acid could be used as a natural bactericide for the control of bacterial wilt in tobacco.

In this study, we confirmed that the accumulation of caffeic acid was significantly increased when applied to tobacco plants challenged by *R. solanacearum*. Meanwhile, caffeic acid inhibited *R. solanacearum* by regulating biofilm formation and colonization in the root. In addition, caffeic acid treatment effectively activated resistance enzyme activities and promoted lignin and hydroxyproline accumulation, thereby inducing the occurrence of tobacco bacterial wilt. These findings may provide theoretical support for the control of tobacco bacterial wilt by caffeic acid.

## Data Availability Statement

The original contributions presented in the study are included in the article/supplementary material, further inquiries can be directed to the corresponding author.

## Author Contributions

SL conceived and designed the experiments, performed the analysis, and wrote the manuscript. JP, LY, and HZ contributed to collecting root exudates and analyzed the data. XZ and WD designed the experiments and modified the manuscript. All authors contributed to the article and approved the submitted version.

## Conflict of Interest

The authors declare that the research was conducted in the absence of any commercial or financial relationships that could be construed as a potential conflict of interest.

## Publisher’s Note

All claims expressed in this article are solely those of the authors and do not necessarily represent those of their affiliated organizations, or those of the publisher, the editors and the reviewers. Any product that may be evaluated in this article, or claim that may be made by its manufacturer, is not guaranteed or endorsed by the publisher.
